# Factors associated with gender equality among church-going young men in Kinshasa, Democratic Republic of Congo: a cross-sectional study

**DOI:** 10.1186/s12939-017-0707-7

**Published:** 2017-12-11

**Authors:** Hendrew Lusey, Miguel San Sebastian, Monica Christianson, Kerstin E. Edin

**Affiliations:** 1 World Council of Churches, Central Africa Regional Coordinator of the Ecumenical HIV and AIDS Initiative in Africa (EHAIA), C/o Salvation Army Headquarter, Avenue Colonel Ebeya no 23, B.P. 8636 Kinshasa Gombe, Democratic Republic of Congo; 2 Department of Public Health and Clinical Medicine, Epidemiology and Global Health, Umeå University, Umeå, Sweden; 3 Department of Nursing, Sexual and Reproductive Health, Umeå University, Umeå, Sweden

**Keywords:** Gender equality, Gender-equitable men scale, Church-going young men, Masculinities, Cross-sectional study, DR Congo

## Abstract

**Background:**

While women and girls are made vulnerable by inequitable and violent versions of masculinities, there is increasing evidence that gender equality will not be achieved without partnering with men. The aim of this study was to assess gender-equitable norms and their determinants among church-going young men in Kinshasa, the Democratic Republic of Congo.

**Method:**

A cross-sectional study was carried out among 289 church-going young men, aged 18–24 years, residing in three disadvantaged communes of Kinshasa. Variables included sociodemographic characteristics, attitudes towards gender equality and responses to issues related to the Gender-Equitable Men (GEM) scale. Logistic regression was applied to identify the associations between sociodemographic characteristics, attitudes and the GEM scale.

**Results:**

The findings provide evidence of attitudes and beliefs that act as barriers to gender equality. For instance, the majority of church-going young men (83.74%) agreed that a man is the only decision maker in the home and about half (50.87%) of the respondents supported the statement “There are times a woman deserves to be beaten”. Similarly, around half of the participants agreed with the idea of men’s uncontrollable sex drive (50.87%) and men’s toughness (50.17%). Close to half of the participants (44.29%) agreed that it is women’s responsibility to prevent pregnancy. These attitudes co-existed with a few gender-equitable norms as 82.70% agreed on the importance of joint decisions concerning family planning. An association between education, certain places of residence, being single or separated, and supportive attitudes towards gender equality was found with higher scores for the GEM.

**Conclusion:**

Our study findings indicate that a high proportion of church-going young men do not endorse gender-equitable norms. Therefore, churches urgently need comprehensive gender equality and masculinity policies and programmes to influence young men’s attitudes and behaviours. The promotion of gender equality in schools and the wider community also need to be encouraged**.**

## Background

### Youth masculinities and health

Policies and programmes that equate gender dimensions of health solely with the specific health needs of women have failed to address the evidence of gendered determinants that affect the health of both men and women [1]. Gender equality work − concerning the equal rights, responsibilities and opportunities of women, men, girls and boys − has tended to focus on the needs of women and girls. However, there is increasing recognition that gender equality will not be achieved without engaging men and boys as partners in the process [2]. Over the last decades, numerous programmes have sought to work with men and boys to challenge harmful norms of masculinities − what it means to be men [3] − with the purpose of improving the health of women, children and men. Some men and boys have significantly contributed to promoting gender-equitable attitudes and behaviours after programmatic interventions on masculinities [4]. For instance, after the implementation of some of such interventions in Brazil, India and Ethiopia, young men successfully changed their attitudes related to dominant versions of masculinities, particularly with regard to the negative consequences of actions that had made them and their partners vulnerable to HIV [5–7].

Nevertheless, inequitable norms of masculinity − those beliefs and expectations regarding positions and behaviours deemed appropriate for men and boys in a given society − may still put their health and that of their partners in jeopardy [8]. For instance, young men who internalise norms that emphasise sexual prowess and conquest as crucial in being a man [9] may have sex with multiple partners where protection is not an issue of concern [10]. They may also think that making a woman pregnant validates what it means to be a man [11] and may otherwise consider pregnancy prevention as the woman’s responsibility [12]. Research suggests that young men may learn to view women as sexual objects and may use coercion to engage in sexual activities [13]. These beliefs and inequitable attitudes may begin in adolescence and may continue into adulthood in the absence of adequate spaces for reflection [14]. Therefore, engaging young men effectively in thinking critically about inequitable norms and taking concrete actions to promote gender equality can lead to better, longer, healthier lives and more fulfilling relationships for the young men themselves, as well as young women and children [15].

### Masculinities in sub-Saharan Africa and the positions of churches

In sub-Saharan Africa **(**SSA), HIV is still a very serious public health problem as two thirds of people living with HIV reside in the region and some men are portrayed as key drivers [16]. The HIV epidemic and its implications for the population’s health have motivated churches to address, among other approaches, issues of masculinities [17]. Traditionally, many African churches have been instrumental in addressing HIV prevention in accordance with the strategic priorities of the World Health Organization (WHO) and primary health care principles [18]. Churches have also learnt many lessons from HIV and AIDS responses that have been applied in the field of masculinities. For instance, a study has indicated that certain African churches could be contributing progressively to the transformation of harmful norms of masculinities [19]. Therefore, given the population coverage, churches in Africa may provide a compelling setting and a powerful socialisation platform for engaging large numbers of youth with positive attitudes and behaviours towards gender equality and with discourses such as those supporting positive forms of masculinities [20].

However, some church leaders’ traditional views regarding what it means to be a man may reinforce harmful norms of masculinities that are prevalent in Africa [21]. Dominant versions of masculinities may partly be the result of selective reading and interpretation of the scriptures [22], lack of church leaders’ commitment to promoting gender equality [23], and their real and/or perceived complacency in condoning harmful beliefs and practices about what it means to be a man [24]. For instance, a study found significant gender differences in many Mozambican churches, in which condom use was more likely to be discussed in men’s rather than at women’s meetings [25]. In spite of this, some church youth report that churches are very important to them and they still believe in the church message regarding masculinity issues [26]. Therefore, churches have an opportunity to explore and identify harmful beliefs, attitudes and practices regarding youth masculinities and work to overcome them [27].

### Masculinity studies in the Democratic Republic of Congo (DRC)

The majority of Congolese consider themselves as believers, which include Catholic (31%), Protestant (30%), other Christian churches (34%), indigenous religions (3%) and Muslims (2%). The church seems to play an important role in Congolese life and understanding how the church impacts on masculinity is necessary for designing an effective promotion of gender equality. The Ecumenical HIV and AIDS Initiative in Africa (EHAIA) has been strengthening the capacity of local church leaders by providing them with training and support to work with men and boys as part of the solution in transforming negative norms of masculinities and responding to HIV. A qualitative interview study conducted in the DRC indicated that the EHAIA’s focus on masculinities resulted in some church youth embracing gender equality values (care, responsibility and nonviolence) as alternative ways of being a man [26].

With a focus on understanding men’s practices and attitudes related to gender equality, the International Men and Gender Equality Survey (IMAGES) explored attitudes concerning gender relations, gender-based violence and the effects of conflict on women and men in Eastern DRC [28]. The IMAGES data suggested that both Congolese men and women ascribe to unequal gender norms that allow men to be sceptical about gender equality, while women have internalised these gender norms, transforming them into oppressed citizens. The same study also suggested that inequitable gender attitudes significantly affect women’s sexual relationships. For instance, 62% of women and 48% of men surveyed said that a man has the right to sex even if a woman refuses [28]. Despite the invaluable DRC-IMAGES, there is a lack of masculinity and gender equality-related studies in a church-based environment and specifically among church youth, both internationally and in the DRC. In an attempt to fill this knowledge gap, this study aimed to assess gender-equitable norms and their determinants among church-going young men in Kinshasa, the capital city of the DRC.

## Methods

### Study setting

This study was conducted in Bumbu, Camp Luka and Masina, 3 disadvantaged peri-urban communes out of the 24 situated in Kinshasa. The city has a population of nearly 12 million of a total population of 77 million in the DRC [29]. A convenient sample of three communes was selected because of the work that the Salvation Army and Eglise du Christ au Congo have developed with youth in the study settings and the collaboration between the first author and the leaderships of these churches. In this regard, the first author has been building the capacity of church leaders and church youth through masculinity workshops to equip them with the knowledge and skills to identify and fill gaps in male engagement programming related to HIV prevention, care, treatment and support. This has enabled the development of relevant and effective programmes and policies that have engaged men and young men in the responses of the church to HIV.

### Variables

The overall questionnaire included 50 structured questions that were organised in three groups: sociodemographic characteristics, attitudes towards gender equality and the gender-Equitable Men (GEM) scale [30]. Certain items related to gender equality were used from the IMAGES project [30], one of most the comprehensive studies carried out in more than 20 countries (including the DRC) regarding men’s practices and attitudes to gender norms, attitudes to gender equality policies, household chores, intimate partner violence, health and economic stress. Given the focus of our study on gender equality, health and economic stress were deliberately omitted. In our study, a few items regarding certain church aspects, such as church belonging, church attendance and attendance frequency were added to the sociodemographic characteristics.

#### Sociodemographic characteristics

Age was categorised in three groups (18–19, 20–21 and 22–24 years) and civil status included single, married/cohabiting and separated. Education comprised three levels: primary, secondary and university. Information on place of residence and church affiliation was also collected. Church attendance frequency had three categories: always, often and seldom. The main source of income included heads of households and young men’s income. Labour status was dichotomised into employed and unemployed/students.

#### Attitudes towards gender equality

With regard to young men’s perspectives on gender equality, six questions based on the IMAGES questionnaire were posed [31]. Responses to these questions fell into four categories, as participants were asked to ascertain whether they completely (1) or partly agreed (2) or they partly (3) or completely disagreed (4) with the statements. An index created by the sum of all the items was developed and divided in tertiles, representing low, medium and high levels of attitudes toward gender equality. The responses of church-going young men to the attitudes stated in the questionnaire are reported in Appendix.

#### The gender-equitable men (GEM) scale

Originally, the GEM scale was developed in response to the relative lack of tools required to measure quantitatively changes resulting from gender-related interventions [32]. The GEM scale resulted from qualitative research on gender norms with young men in low-income settings in Brazil, but has been validated in several contexts, including India, Kenya, Uganda and Nicaragua, as well as being adapted for the Ethiopian context [14]. The GEM scale has 24 items related to gender and domestic life, violence against women, sexual relationships, masculinities and sexual and reproductive health. In our study, the GEM questionnaire included a list of 16 statements only and we did not select the 8 remaining that we considered as irrelevant for young people. The participants were requested to indicate their extent of agreement (total agreement, partial agreement or disagreement) with a list of statements. The items were summed up and first grouped in tertiles for descriptive purposes and later dichotomised using the mean as the cut-off point for low and high GEM for the regression analysis.

### Recruitment and data collection

This cross-sectional survey was conducted from March to April 2016. The first author received the French translated questionnaire from Promundo, which is a nongovernmental organisation founded in Brazil aimed at promoting caring, non-violent and equitable masculinities and gender relations [28]. The questionnaire was pre-tested among 10 young men from a church in a different but similarly disadvantaged commune of Kinshasa to the study participants. The pre-test sought to determine whether or not these items were clear, understandable and culturally relevant to the Congolese church youth context. In general, the participants considered the questionnaire appropriate, except for a few items that were re-worded by the study team. We reworded these items because we perceived them as inappropriate for the Congolese church youth context. For instance, one item was originally labelled as follows: “I would be outraged if my wife asked me to use a condom”. Since most of the participants were single, we replaced the word “wife” by “partner.”

To be included in this study, the participants had to be young men, aged 18–24 years, belonging to the Salvation Army or the Eglise du Christ au Congo, living in the above three communes and volunteering to participate. The Revival Churches of Congo were also contacted to participate but the leadership delayed its response and they were not included in the study. Once the study authorisation had been granted by the leadership of both churches involved, the first author contacted the respective pastors at the parish levels, who identified and recruited young men from local parishes, particularly on Sundays. When these pastors gathered the young men, the first author joined in to provide the self-administered questionnaire to the participants. These participants answered the questionnaire in church premises where the risk of disturbance was relatively low and they took approximately 1 h to complete the questionnaire.

In some cases, particularly for respondents who felt that certain statements were difficult for them to understand, the first author read each question to the respondent and then the respondent ticked the answers on the printed form of the questionnaire. The sample size calculation was based on the baseline prevalence of GEM of 50% as reported in other studies [6] and at the 5% level with 80% power resulting in 370 individuals. In total, 400 young men were invited to participate, 50 were excluded for not meeting the inclusion criteria (including not being in the appropriate age range for the study, residing outside the study settings at the time at which data were collected, and belonging to a church other than the two selected), 50 did not show up and 11 declined the invitation for personal reasons. Finally 289 young men, who met the inclusion criteria, completed the questionnaire.

### Data analysis

The survey responses were recorded on paper questionnaire forms, then entered into an Excel spreadsheet and later transferred into STATA 13.1 for statistical analysis. First, the percentages of responses related to the sociodemographic characteristics of the young men, attitudes towards gender equality and GEM were calculated. After that, logistic regression analysis was conducted to assess the relationships between the sociodemographic characteristics and the gender equality attitudes of the participants using the GEM scale. Crude and adjusted odds ratios and their 95% confidence intervals were calculated. Significant variables in the crude model were included in the adjusted models.

### Ethical approval

This study was approved by the institutional review board of the School of Public Health at the University of Kinshasa. Before doing the fieldwork, the first author requested written permission for the study from the Salvation Army and the Eglise du Christ au Congo leaderships. Also, the aim and purpose of the study were explained to the young men participating, who were ensured anonymity. Written informed consent was obtained from all the study participants.

## Results

Table 1 presents the sociodemographic characteristics of the 289 young men (18–24 years old) of whom 55.36% were aged 18–19 years. The majority of the respondents (65.40%) were single and had secondary school level education (56.40%). The sample was equally distributed among the three communes. While most of these participants (91.70%) were from the Salvation Army, only 8% belonged to the Eglise du Christ au Congo. In the group of participants, 77.16% were church attendants and 40.83% identified themselves as regular church attendants. When asked about their main reason to be in the church premises on the day of data collection, more than half (54.41%) reported that they were attending the Sunday church services. While the main sources of income were from parents 73.70%, almost one fourth (22.49%) identified themselves as employed. Around one fifth (20.07%) of church-going young men scored low in the gender attitude questions, with the majority (61.94%) scoring moderate and another fifth (17.99%) scoring high. Close to four out of ten (38.75%) showed low support for inequitable gender norms, agreeing with statements of gender inequality, a third (30.10%) had moderate scores for GEM (partly agree) and another third (31.14%) had high scores (strongly agree).The majority of young men supported low and moderate gender equitable norms and only one third endorsed high GEM scores, meaning positive norms towards gender equity**.**

**Table 1:** Sociodemographic characteristics, attitudes towards gender equality and GEM scores of church-going young men in Kinshasa

	*n* (%)
Age group	
18–20	160 (55.36)
21–22	67 (23.18)
23–24	62 (21.45)
Marital status	
Married/cohabiting	64 (22.15)
Single	189 (65.40)
Separated	36 (12.46)
Highest grade	
Primary school	27 (9.34)
Secondary school	163 (56.40)
University	99 (34.26)
Place of residence	
Bumbu	105 (36.46)
Masina	97 (33.68)
Camp Luka	86 (29.86)
Church affiliation	
Salvation Army	265 (91.70)
Eglise du Christ au Congo	24 (8.30)
Church attendance	
Yes	223 (77.16)
No	66 (22.84)
Attendance regularity	
Always	118 (40.83)
Often	73 (25.26)
Seldom	98 (33.91)
Visit purposes	
Sunday church service	158 (54.41)
Bible studies	58 (20.57)
Youth meetings	73 (25.26)
Main source of income	
Young men themselves	72 (24.91))
Heads of households	217 (75.09
Labour status	
Unemployed/students	224 (77.51)
Employed	65 (22.49)
Attitudes to gender equality	
Low	58 (20.07)
Medium	179 (61.94)
High	52 (17.99)
GEM	
Low	112 (38.75)
Medium	87 (30.10)
High	90 31.14)

Table 2 shows the frequency of responses to the 15 questions included in the GEM scale. Overall, many participants (50–60%) responded “totally agree” to all the items, highlighting that the study participants supported several inequitable gender norms. For instance, the majority (66.09%) agreed that a woman’s most important role is to care for her home and suggested changing diapers and feeding children were the mother’s responsibility (64.05%). Around half (50.87%) of the young men agreed that men need more sex than women and (41.81%) were of the opinion that men do not talk about sex, they just do it. The belief that men are always sexually ready, a sign of male virility, was also the subject of agreement for 45.33% of the respondents. More than half (52.94%) of the participants concurred that men should be embarrassed if they unable to attain an erection. In addition, 44.29% of the young men were of the view that women were solely responsible for preventing pregnancy.

**Table 2:** Church-going young men’s scores on the Gender-Equitable Men (GEM) scale

Survey statements	Responses	*n* (%)
A woman’s most important role is to take care of her home and cook for her family	Totally agree	191 (66.09)
	Partially agree	49 (16.96)
	Disagree	49 (16.96)
Men need more sex than women	Totally agree	147 (50.87)
	Partially agree	93 (32.18)
	Disagree	49 (19.86)
Men don’t talk about sex; you just do it	Totally agree	119 (41.18)
	Partially agree	87 (30.10)
	Disagree	83 (28.72)
There are times when a woman deserves to be beaten	Totally agree	147 (50.87)
	Partially agree	85 (29.41)
	Disagree	57 (19.72)
Changing diapers, giving a bath, and feeding kids is the mother’s responsibility	Totally agree	185 (64.05)
	Partially agree	53 (18.34)
	Disagree	51 (17.65)
It is a woman’s responsibility to avoid getting pregnant	Totally agree	128 (44.29)
	Partially agree	99 (34.26)
	Disagree	62 (21.45)
A man should have the final word about decisions in his home	Totally agree	242 (83.74)
	Partially agree	29 (10.03)
	Disagree	18 (6.23)
Men are always ready to have sex	Totally agree	131 (45.33)
	Partially agree	96 (33.22)
	Disagree	62 (21.45)
A woman should tolerate violence to keep her family together	Totally agree	109 (37.52)
	Partially agree	60 (20.76)
	Disagree	120 (41.52)
I would be outraged if my partner asked me to use a condom	Totally agree	137 (47.40)
	Partially disagree	87 (30.10)
	Disagree	65 (22.49)
A man and a woman should decide together if they want to have children	Totally agree	239 (82.70)
	Partially agree	28 (9.69)
	Disagree	22 (7.61)
I would never have a gay friend	Totally agree	166 (57.44)
	Partially agree	53 (18.33)
	Disagree	70 (24.22)
If someone insults me, I will defend my reputation, with force if I have to	Totally agree	116 (40.28)
	Partially agree	91 (31.60)
	Disagree	82 (28.12)
To be a man, you need to be tough	Totally agree	145 (50.17)
	Partially agree	85 (29.41)
	Disagree	59 (20.42)
Men should be embarrassed if they are unable to get an erection	Totally agree	153 (52.94)
	Partially agree	68 (23.53)
	Disagree	68 (23.53)

A significant proportion (47.40%) of the young men indicated that they would be outraged if their partners asked them to use a condom. They also reported homophobic attitudes, with 57.44% declining a friendly relationship with a gay man. Half (50.17%) of the young men agreed with the statement that men should be tough and most of them (83.74%) also concurred that men have the final word concerning decisions in their homes. Nearly half (47.40%) agreed that they would defend their reputation with force if they had to, for example if someone insulted them. In contrast, the same young men also tended to support certain equitable gender norms, with most (82.70%) agreeing that a man and a woman should decide together if they want children. Similarly, nearly half (41.52%) disagreed with the statement that women should tolerate violence to keep the family together.

Table 3 presents the logistic regression model for factors associated with high support (totally agree) on the GEM scale among young male churchgoers. In the crude analysis, several factors were significantly related to a higher GEM score. In the multivariate regression model, those who lived in the communes of Camp Luka and Masina also had higher odds on the GEM scale compared to those living in Bumbu and being single or separated was statistically significantly associated with high GEM values. A clear gradient in the association between attitudes to gender equality and the GEM scale was found, with those with more equal attitudes having higher odds on the GEM scale. Although education was not significant, higher GEM values were found among those with secondary education and above compared to those with primary education. Similarly, though the association was not significant, men who always attended church services had higher odds of high GEM scores compared to those who seldom attended the church.

**Table 3:** Factors related to high GEM reported by church-going young men: logistic regression analysis with crude and adjusted odds ratios (OR) and their 95% confidence intervals (CI)

Factors	Crude OR (95% CI)	Adjusted OR (95% CI)
Age		
23–24	1	–
21–22	0.56 (0.28–1.12)	–
18–20	1.02 (0.57–1.84)	–
Education		
Primary school	1	1
Secondary school	3.04 (1.22–7.58)	2.65 (0.99–7.11)
University	3.43 (1.33–8.84)	2.54 (0.90–7.16)
Place of residence		
Bumbu	1	1
Camp Luka	2.53 (1.41–4.56)	2.27(1.18–4.38)
Masina	3.10 (1.75–5.52)	3.29 (1.73–6.25)
Civil status		
Married/cohabiting	1	1
Single	2.75 (1.51–5.02)	3.20 (1.64–6.25)
Separated	2.75 (1.18–6.39)	3.54 (1.36–9.21)
Job status		
Employed	1	–
Unemployed	1.05 (0.78–1.40)	–
Church affiliation		
Salvation Army	1	–
Eglise du Christ au Congo	0.69 (0.30–1-60)	–
Church attendance		
No	1	−
Yes	1.07 (0.61–1.85)	–
Attendance frequency		
Seldom	1	–
Often	1.02 (0.55–1.88)	–
Always	1.49 (0.87–2.56)	–
Main sources of income		
Young men	1	–
Parents	1.46 (0.85–2.50)	–
Attitudes to gender equality		
Low equity	1	1
Medium equity	2.00 (1.07–3.76)	2.56 (1.30–5.04)
High equity	9.33 (3.84–22.63)	7.09 (2.81–17.89)

## Discussion

Our research findings show attitudes and beliefs that may act as potential barriers for gender equality among church-going young men. They scored highly on gender-inequitable norms concerning statements regarding attitudes related to gender and domestic life, violence against women, what it means to be a man, sexuality and reproductive and sexual health. In spite of this, our study also offers some hope for change as participants also tended to support a few equitable gender norms. An association between education, certain places of residence, being single or separated, and supportive attitudes towards gender equality was found with higher scores on the GEM scale. In the following section, first we consider the GEM dimensions and then compare our findings to those of similar studies. Finally, we offer some reasons for the differences and discuss the results of the regression analysis.

### GEM domains

#### Gender and domestic life

Research suggests that men may strongly adhere to unequal gender norms, holding women responsible for domestic duties and child care and viewing men as the final decision makers in the home [26, 28, 32, 33]. Most (74.5%) of the men in DRC-IMAGES [28] agreed that “a woman’s most important role is to take care of her home and cook for her family”, reflecting a similar proportion in a study in Rwanda (83.1%) [33] and our own study (83.05%) [31, 33]. Similarly, our findings show a higher reluctance among young men to participate in child care, with 83.05% agreeing that changing diapers, feeding and giving children a bath are a mother’s responsibility, compared to 53.3% in DRC-IMAGES [28] and 61.2% in Rwanda [31, 33]. We found also a strong emphasis on the traditional positions of men as the head of the household. This was depicted in the belief that a man should have the final word about decisions in his home, 93.77% in our study compared to 75% in DRC-IMAGES [28], 65.9% of men in Rwanda and 80% in India [31, 33].

#### Violence against women

Studies have shown that respondents who provide support for inequitable gender norms are also more likely to use physical and sexual violence against their partners [32]. While having similar prevalence with other studies [28, 31, 33], with rates of 60–65% regarding the statement: “A woman should tolerate violence in order to keep her family together”, 80.21% of our study participants strongly agreed that “there are times when a woman deserves to be beaten”, compared to the DRC-IMAGES (61.9%) [28] and studies in India (64.4%) [33] and South Sudan (63%) [34]. These gender inequitable norms prevailing in the DRC may attest to the reasons why this country has the highest rates of intimate partner violence and sexual violence compared to other countries in which IMAGES projects have been conducted [28].

#### Sexual relationships

Gender norms that dictate what is appropriate or expected regarding sexual behaviours for men and women may encourage risk behaviours, particularly among young men [35]. An example of such gender norms is the view that men need more sex than women, a view endorsed by 67.83% of church-going young men, similar to DRC-IMAGES (70.5%) [28]. The belief that men are always ready to have sex was held by 78.55% of our study participants, 65% of men in DRC-IMAGES [28] and 46% in a study conducted in Ethiopia [7]. These findings, including ours, suggest that Congolese men strongly adhere to unequal gender norms and tend to be sceptical concerning gender equality as they think that they have a “right to have sex”, particularly with their female partners, even if their partners refuse [28].

#### Masculinities

Research in African contexts has shown that masculinity ideals give centrality to toughness, strength and heterosexual performance [36]. In various studies, the majority of men (79.58% in our study, 85.8% in a GEM study in India and 73% in Bosnia) concurred that to be a man you need to be tough [33]. This may suggest the pressure facing young men to exert power and authority over women and other men to gain praise and recognition for their masculinities [37]. In addition, a study in Uganda has documented stereotypes defending reputation and strength being equated with what it means to be a man for fear of social embarrassment [38]. In this regard, 71.88% of our study participants, compared to 55.3% in DRC-IMAGES [28] and 91.7% in India [31, 33], were of the view that if somebody insulted them, they would defend their reputation, with force if they have to. Research indicates that homophobia is perceived as an expression of masculinity [39]; indeed, 75.77% of our study participants and 65% in DRC-IMAGES [28] would deny a friendship with a gay man. Our findings are consistent with research in India and Bosnia, reporting that men feel uncomfortable (92% and 56.8% respectively) when in contact with a gay man [40].

#### Sexual and reproductive health

Social norms and attitudes which put men in a position of sexual dominance may be related to unsafe sexual behaviours with potential negative consequences inhibiting women from controlling their own sexual and reproductive health [14]. These norms dictate that it is men’s responsibility to acquire condoms as a woman carrying condoms may be viewed as “easy”. Moreover, as many as 77.50% of the young men in our study, 66.4% in DRC-IMAGES [28] and 52% in a Tanzanian study [41] agreed that they would be outraged if their partner asked them to use a condom. In many settings, the prevailing norms indicate that sexual and reproductive health is perceived as “a female domain” and therefore it is a woman’s responsibility to avoid getting pregnant [12, 42]. In our study 78.55% agreed with this statement versus 61% in DRC-IMAGES [28, 43]. Yet, gendered power dynamics may prevent certain women from negotiating condom use even when they wish to do so [33]. In spite of these persistent inequitable attitudes, our respondents emphasised the importance of mutual family planning. For instance, 92.39% of church-going young men agreed that a man and a woman should decide together if they want children.

Compared to DRC-IMAGES [28] and GEM findings from other studies carried out in Brazil, Chile, Croatia, India, Mexico and Rwanda [31, 33, 44], church-going young men in our study setting in DRC mainly scored inequitable statements, which could be due to several reasons. Research suggests that social expectations of what it means to be a man might remain reinforced at individual and community levels for young men who live in areas where they may have few opportunities to challenge their beliefs and attitudes with those modelling alternative masculinities [28]. Another study indicated that young men from disadvantaged areas and backgrounds face many challenges, including lower levels of health-promoting behaviours, poor health outcomes and lack of social support from parents and friends [45]. Therefore, church-going young men living in disadvantaged areas such as in our study may continue to be influenced by the same harmful norms of masculinity and socioeconomic health inequalities as the rest of society supporting inequitable norms [17].

In addition, research has highlighted that some Christian views and negative cultural aspects regarding patriarchy tend to reinforce each other in certain churches, which may provide considerable power to men and young men [43, 46]. Some churches may even reinforce harmful norms of masculinity through skewed teachings and interpretations of the scriptures and these may be embraced by church-going young men [22]. In this regard, a masculinity-related study conducted in Zambia found church-going young men who referred to the Genesis creation story as evidence of male superiority [47]. This is an indication that scriptures may be used and abused by young men to engage in risky sexual behaviours and to display oppressive attitudes towards women and girls.

The Salvation Army is a progressive church in this regard since priesthood vocation is open and accessible to both women and men. However, we still agree that unequal gender relations and opinions might exist between priest women and men [21]. Moreover, the church efforts that have been undertaken so far by the Ecumenical HIV and AIDS Initiative in Africa in this study population to promote positive forms of masculinities and gender equality might have reached only a small proportion of church-going young men [26]. While some church hierarchies and policies might express progressive views on gender equality, members of some congregations might not endorse egalitarian attitudes and relations. Research shows that masculinity and gender equality issues might be fairly new subjects for some Congolese churches, which thus might not have effective policies and programmes addressing such issues [26].

### Determinants of gender-equitable norms by men

Logistic regression models were used to identify associations between sociodemographic characteristics, attitudes to gender equality and the GEM scale. The regression analysis showed that being educated, living in Camp Luka and Masina, being single or separated, and having supportive attitudes towards gender equality were significantly associated with high support for GEM. Although education was not statistically significant in the adjusted model, probably due to the small sample size, the results seem to point to the importance of formal education. Research has shown that educated men exposed to broader ideas about gender may socialise with other people, expand their minds and question certain norms regarding what it means to be a man. In addition, they may have progressive views of gender equality and may be aware of its importance [40].

Another important set of findings in our study was that young men living in Camp Luka and Masina and those who were single or separated had high GEM scores. The association between living in Camp Luka and Masina and high support for GEM was unexpected as the areas selected for this study shared similar patterns in terms of sociodemographic characteristics and church affiliations. In contrast, the observed association between being separated and high support for GEM was found in DRC-IMAGES [28], in which unmarried and non-cohabiting men with children were more likely to report increased participation in child care [33]. This might be an indication that these men spend quality time with their children outside their mothers’ homes and/or in the absence of their mothers [28]. Qualitative studies may be needed to gain a better understanding of such differences in both cases.

In our study, the strongest association was found between supportive attitudes towards gender equality and high scores for GEM (strongly agree). This was expected as research using the GEM scale has shown how working with men and boys to promote gender equality can contribute to achieving positive forms of masculinities [48]. These include less oppressive, non-violent, caring and equitable forms of masculinities progressively emerging in SSA and elsewhere [49]. The IMAGES projects carried out in Brazil, Chile, Croatia, India, Mexico and Rwanda have confirmed these findings [31]. Similarly, other study findings [31, 33], including ours, indicate that men with equitable attitudes are more likely to report an active participation in household duties, effective communication with their female partners and reduced use of violence.

Despite the lack of statistical association, probably due to the relative small sample size, young men who always attend church services had a better GEM score than those who rarely attended. Though more work needs to be done, this might imply that churches have the potential to support gender equitable norms among young men. This finding is consistent with that of a masculinity and religion study in Nigeria suggesting that supportive attitudes towards gender equality are consistent with certain church teachings on masculinity issues [50]. In this vein, a masculinity study carried out in the Assembly of God church in Zimbabwe found that men who were taught to be loving and non-violent in relationships with their female partners exhibited mutual support [51]. Similar studies have shown that some churches may be progressively contributing to changing gender norms, building and strengthening healthy family ties for men to partner constructively with their spouses and achieve gender equality [21, 52]. However, we are aware that not all churches are the same and not all pastors in the churches share similar perspectives regarding gender and sexuality.

### Study strengths and limitations

Our study has a number of strengths, including the attempt to provide a general overview of church-going young men’s beliefs and attitudes regarding the determinants of gender equality in marginalised areas in the DRC. The collaboration between the pastors and researchers contributed to reaching this vulnerable and under-researched population. Our study is not without limitations as the relative small sample size could not have identified some important relationships using logistic regression models. The views expressed by the respondents who mainly came from the Salvation Army might not have been representative of all-church going young men attending other churches in Kinshasa or in the rest of the country. Other possible limitations consisted in the fact that the study was based on self-reported attitudes and behaviours and the reliability of the responses could not be assessed and a potential bias might have taken place when parish pastors selected the participants.

Due to misunderstandings among participants about some statements, the survey tool was not self-administered for a few participants, potentially leading respondents to offer socially desirable responses. However, given the negative attitudes to equitable gender norms that church-going young men displayed, the interview techniques might not have significantly influenced their answers to please the investigator. Although the attitude and GEM questionnaires have been previously validated and extensively used in African contexts, we are aware that some statements might not have been fully understood in the same way by all the study participants, introducing a certain response bias.

The first author is the regional coordinator of EHAIA, a project of the World Council of Churches and is active in masculinity and gender equality work. Respondents may have provided responses to impress him (social desirability bias). Participants were encouraged to stick on statements of their choice and were ensured anonymity. The first author considered himself as having two perspectives: insider (working for a church) and outsider (as a researcher) in the research process [53]. He tried to manage these two perspectives by remaining a curious, open-minded and interested observer alongside the survey. The first author conducted this study in close collaboration with a study team of researchers that had different professional and national backgrounds. This collaboration process required several critical discussions and interpretations of the findings during the analysis, which resulted in agreements that enhanced data credibility.

## Conclusions

To the best of our knowledge, this is the first study to measure views concerning gender norms using the GEM scale and some selected questions related to gender attitudes from IMAGES with church-going young men. Our study findings indicate that church-going young men are highly supportive of inequitable gender norms. However, being educated, living in Camp Luka and Masina, being single or separated, always attending the church and endorsing gender equitable attitudes were found to be key determinants associated with high scores on GEM. Our study calls attention to the need to involve church-going young men early in life in addressing inequitable attitudes, gender norms and deep-rooted ideologies of masculinities that it may be difficult to transform later during adulthood. Church-going young men with alternative views may present a key opportunity to reflect upon, challenge and change inequitable gender norms when they are equipped to do so before they may internalise societal messages that dictate (often negative) behaviours deemed appropriate for men. The inclusion of church-going young men as an indispensable part of gender equality and masculinity programmes in churches requires an approach that includes them not just as individuals but also as a collective. More intense promotional efforts are required as part of public education, early adolescent education and church policy and programme development related to gender equality. Further studies could be warranted to explore the determinants of gender equality from both female and male church-going youth’s perspectives to ascertain any gender differences in this regard.

## Appendix

**Table 4:** Table regarding attitudes toward gender equality related to relations between men and women among church-going young men in Kinshasa

Statements	Completely agree	Partly agree	Partly disagree	Completely disagree
When women work, they are taking jobs away from men	109 (37.72)	57 (19.72)	25 (8.65)	61 (21.11)
When women get rights, they are taking rights away from men	91 (31.49)	71 (24.57)	42 (14.53)	55 (19.03)
Rights for women mean that men lose out	51 (17.65)	41 (14.19)	44 (15.22)	101 (34.95)
When a woman is raped, she usually did something careless to put herself in that situation	91 (31.49)	71 (24.57)	52 (17.99)	47 (16.26)
In some cases, women actually want it to happen	67 (23.18)	80 (27.68)	38 (13.15)	73 (25.26)
If a woman did not fight back, you can’t really say that it was rape	117 (40.48)	77 (26.47)	31 (10.73)	46 (15.92)
In any case one would have to question whether the victim is promiscuous	122 (42.26)	51 (17.65)	39 (13.49)	57 (19.72)
In any case one would have to question whether the victim has a bad reputation	88 (30.45)	78 (26.99)	44 (15.22)	58 (20.07)
